# Epigenetic regulation of the lineage specificity of primary human dermal lymphatic and blood vascular endothelial cells

**DOI:** 10.1007/s10456-020-09743-9

**Published:** 2020-09-12

**Authors:** Carlotta Tacconi, Yuliang He, Luca Ducoli, Michael Detmar

**Affiliations:** Institute of Pharmaceutical Sciences, Swiss Federal Institute of Technology, ETH Zurich, Vladimir-Prelog-Weg 3, HCI H303, 8093 Zurich, Switzerland

**Keywords:** Blood endothelial cells, Lymphatic endothelial cells, Cell identity, Epigenetics, DNA methylation, Histone modifications

## Abstract

Lymphatic and blood vascular endothelial cells (ECs) share several molecular and developmental features. However, these two cell types possess distinct phenotypic signatures, reflecting their different biological functions. Despite significant advances in elucidating how the specification of lymphatic and blood vascular ECs is regulated at the transcriptional level during development, the key molecular mechanisms governing their lineage identity under physiological or pathological conditions remain poorly understood. To explore the epigenomic signatures in the maintenance of EC lineage specificity, we compared the transcriptomic landscapes, histone composition (H3K4me3 and H3K27me3) and DNA methylomes of cultured matched human primary dermal lymphatic and blood vascular ECs. Our findings reveal that blood vascular lineage genes manifest a more ‘repressed’ histone composition in lymphatic ECs, whereas DNA methylation at promoters is less linked to the differential transcriptomes of lymphatic versus blood vascular ECs. Meta-analyses identified two transcriptional regulators, BCL6 and MEF2C, which potentially govern endothelial lineage specificity. Notably, the blood vascular endothelial lineage markers CD34, ESAM and FLT1 and the lymphatic endothelial lineage markers PROX1, PDPN and FLT4 exhibited highly differential epigenetic profiles and responded in distinct manners to epigenetic drug treatments. The perturbation of histone and DNA methylation selectively promoted the expression of blood vascular endothelial markers in lymphatic endothelial cells, but not vice versa. Overall, our study reveals that the fine regulation of lymphatic and blood vascular endothelial transcriptomes is maintained via several epigenetic mechanisms, which are crucial to the maintenance of endothelial cell identity.

**Electronic supplementary material:**

The online version of this article (10.1007/s10456-020-09743-9) contains supplementary material, which is available to authorized users.

## Introduction

The lymphatic and the blood vasculature exert complementary functions in humans. While the blood vasculature represents a closed circulatory system essential for the delivery of oxygen and nutrients, the lymphatic circulation is responsible for draining interstitial fluid from peripheral tissues, also serving as a conduit for immune cell trafficking and lipid absorption in the gut [1]. Both types of vasculature form highly branched networks that are lined by endothelial cells (ECs). Blood capillaries, which are characterized by continuous inter-endothelial junctions, are surrounded by pericytes embedded in a basement membrane. In contrast, lymphatic capillaries are thin-walled, highly permeable blind-ended vessels that lack pericyte coverage and a continuous basement membrane. These capillaries converge to form larger collecting vessels surrounded by mural cells. Collecting lymphatic vessels are furnished with intraluminal valves that enable the unidirectional transport of lymph back to the blood circulation through the lymphovenous valve junctions located around the jugular region [2].

Despite distinct functions of the blood vascular and the lymphatic endothelia, they share a close developmental relationship. During early mammalian embryonic development, endothelial progenitors differentiate from the mesodermal tissue to form a primitive vascular system. This ancestral vascular plexus then undergoes remodeling, resulting in endothelial sprouting (angiogenesis) and specification into arterial or venous cell fates. After the formation of arteries and veins, the first lymphatic ECs (LECs) transdifferentiate from the cardinal vein [3]. Lymphatic development occurs in a stepwise process, where a subset of ECs starts to express COUP-TFII and SOX18, sequentially inducing the expression of the prospero-related homeobox 1 transcription factor (PROX1) [4]. PROX1 is considered to be one of the “master regulators” during lymphatic development and it is the PROX1-positive cells that commit to the lymphatic lineage [5, 6]. Upon terminal differentiation, LECs express several lineage-specific markers, including PROX1, the transmembrane glycoprotein podoplanin (PDPN) and the vascular endothelial growth factor receptor 3 (VEGFR-3 or FLT4) [7, 8]. The conservation of EC identities is crucial for specialized vasculature functions. However, ECs retain, at least experimentally to some extent, the capacity to transdifferentiate into the alternate endothelial lineage, by repression of genes that determine the pre-existing cell fate and/or by activating the opposing markers [9]. Such endothelial plasticity has also been observed under pathological conditions in vivo [10]. For instance, in inflammation and cancer, blood vessels can change phenotypes by expressing lymphatic-specific molecules such as VEGFR-3 [11, 12]. The molecular mechanisms underlying EC differentiation during development have been comprehensively studied, both in terms of marker expression and signaling pathways. However, the mechanisms maintaining EC lineage specificity during postnatal life are still not fully understood.

Activation or suppression of transcription is a highly regulated process that can be orchestrated by epigenetic modifications. Emerging epigenome studies have advanced our understanding of the crosstalk between diverse epigenetic mechanisms and the acquisition of different biological traits during tissue development [13] and under pathological conditions [14, 15]. While recent efforts have been made to uncover the importance of epigenomic architecture for vascular quiescence and specification [16, 17], the epigenetic mechanisms in the maintenance of EC identity remain to be fully elucidated. This has thus fostered our interest in characterizing the epigenetic regulations of the lymphatic versus the blood vascular endothelial lineages. In the present study, we performed comparative analyses of the transcriptomic profiles of blood vascular ECs (BECs) and LECs and of their DNA methylomes and the landscapes of histone modifications. Our results indicate that endothelial lineage-specific markers have specific epigenetic profiles that maintain lineage identity.

## Results

### Distinct transcriptomic patterns of lymphatic and blood vascular endothelial cells

**Fig. 1:**
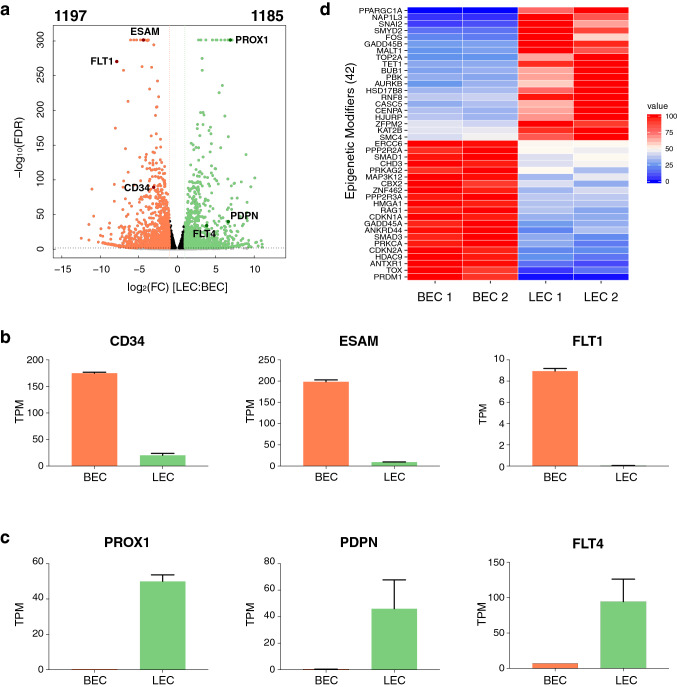
BECs and LECs display lineage-specific transcriptomic landscapes. **a** Volcano plot showing the upregulated genes in BECs (orange) and LECs (green), defined by log2 fold-change (FC) (> 1) and FDR (< 0.01). **b** and **c** Expression levels (in Transcripts Per Million, TPM) of the lineage-specific BEC (CD34, ESAM and FLT1) and LEC markers (PROX1, PDPN and FLT4). **d** Heatmap depicting the expression pattern of the differentially expressed epigenetic modifiers in each cell type. Values are shown as the percentage of maximum expression of the respective gene

We and others have previously reported that BECs and LECs are characterized by distinct expression patterns of lineage-specific markers [18, 19]. To gain a comprehensive view of their differential transcriptomes and to identify key regulatory factors, we first performed deep RNA sequencing of cultured primary human dermal BECs and LECs derived from two individual donors. The purity of isolated cells was confirmed by FACS analysis of the pan-endothelial marker PECAM1 and PDPN. Both LECs and BECs showed high positivity for PECAM1. However, only LECs exhibited a strong positivity for PDPN, while BECs were completely negative for this lymphatic marker (Fig. S1a). The independent biological replicates exhibited high pairwise correlations (Fig. S1b, c). Both cell types conserved high expression levels of the classical EC markers PECAM1 and CDH5 (Fig. S1d). Comparative transcriptomic analysis identified 2382 differentially expressed genes (log2FC > 1 and FDR < 0.01) between BECs and LECs (Fig. 1a). In consistence with the current view of lineage-specific markers, BECs displayed high levels of CD34, ESAM and FLT1 (Fig. 1b), while LECs specifically expressed PROX1, PDPN and FLT4 (Fig. 1c). Differential expression patterns of these lineage-specific markers were further confirmed by qPCR (Fig. S1e, f). Despite recent efforts in depicting the epigenetic landscape of various EC types, very few transcriptomic and DNA methylation signatures pinpointing BEC and LEC identities have been reported [20, 21]. Interestingly, we identified a list of 42 epigenetic regulators, which have previously been described as molecules involved in chromatin remodeling, histone modifications and DNA methylation [22, 23] to be differentially expressed in either EC type (Fig. 1d), implicating a potential role of epigenetic modifications in the maintenance of EC lineage specificity.

### Endothelial lineage specificity is in part regulated at the level of histone modifications

**Fig. 2:**
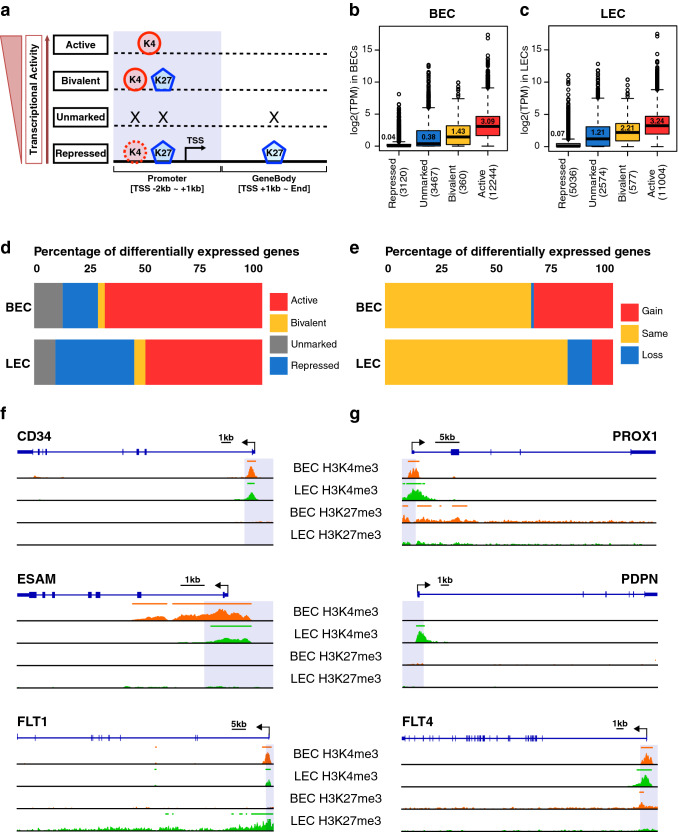
LECs and BECs present different H3K4me3 and H3K27me3 enrichment profiles. **a** Representation of the chromatin composition in each histone state, which was graded by their transcriptional activity. The black arrow labels the transcription start site (TSS) and the direction of the transcription. The promoter region is highlighted in blue. **b** and **c** Expressed genes were categorized into the corresponding histone state in BECs and LECs. Transcriptomic abundances were compared among different histone states and the number of genes in each group is indicated in brackets. The numbers inside the box plots denote the medians. **d** Overview of the histone state of genes with upregulated expression in the respective cell type as determined in Fig. 1a. **e** Proportion of upregulated genes manifesting a sustained or switched histone state, in contrast to the opposing cell type. Differential histone enrichment profiles of the selected BEC (**f**) and LEC (**g**) marker genes. The magnitude of the signal tracks of a given histone modification for a given marker is the same across two cell types. Orange/green bars at the top of each track indicate the called ChIP-Seq peaks

Post-translational modification of histone proteins can markedly affect DNA accessibility to transcriptional regulators. For instance, trimethylation of different lysine residues at the N-terminus may induce either active or repressed chromatin composition [24]. To assess the importance of histone modifications in the maintenance of EC identity, we conducted Chromatin Immunoprecipitation Sequencing (ChIP-Seq) profiling of the H3K4me3 and H3K27me3 histone marks, which are enriched in active or repressed chromatin regions, respectively [25, 26]. Correlation analyses of aligned reads revealed good reproducibility between the two biological replicates (Fig. S2a, b). H3K4me3 was preferentially enriched at promoter regions, while H3K27me3 was more widely spread out and marked distal intergenic regions in a comparable fashion in both cell types (Fig. S2c). To better understand the connection between differential enrichment of histone modifications at promoter regions and the lineage-specific gene expression patterns, we graded the histone enrichment states into 4 levels in an incremental order of transcriptional activity (Fig. 2a). Namely, the ‘repressed’ state denoted by the coverage of H3K27me3 across the gene body or the spanning of H3K27me3 over the promoters in the absence of H3K4me3; the ‘unmarked’ state, where the whole transcript is devoid of any histone marks; the ‘bivalent’ state with both histone modifications residing in the promoter regions; and lastly the ‘active’ state when the promoters are solely enriched for the H3K4me3 mark (Fig. 2a).

The expression level of genes detected in each EC type was in line with the activity of their histone enrichment states (Fig. 2b, c). We then evaluated the histone states of the genes with increased expression in the respective cell type. The majority of upregulated genes were found in an ‘active’ histone state (69% and 51.2% respectively in BECs and LECs), and both cell types showed a similar proportion of upregulated genes in the 'unmarked' or the 'bivalent' state (Fig. 2d). We next investigated if increased gene expression could be attributed to enhanced transcriptional activity of the histone states. We considered the switch of a more favorable histone composition in one cell type compared to the other as a ‘gain’ of transcriptional activity. Genes with the same histone state were also treated as ‘gain’ of activity in the presence of stronger H3K4me3 or weaker H3K27me3 enrichments, and vice versa as a ‘loss’ (Fig. 2e). Surprisingly, only 9.2% of the upregulated genes in LECs resulted from a ‘gain’ of histone states, with another 10.2% showing a ‘loss’ of favorable histone modifications (Fig. 2e). In contrast, a much lower percentage (1.2%) of upregulated genes in BECs displayed a ‘loss’ of histone state (Fig. 2e). Over one third (34.7%) of the genes enriched in BECs had an enhanced transcriptional activity of their histone states, whereas they were conversely maintained at a more ‘repressed’ state in LECs (Fig. 2e, Supplementary Table 1). A closer look at the organization of histone marks of selected lymphatic and blood vascular EC markers revealed that all of them were in the ‘active’ state in the respective cell type. As for the BEC markers, both ESAM and FLT1 acquired a ‘gain’ of histone states in BECs, signified by stronger enrichment and broader coverage of H3K4me3 over the transcription start site (TSS), whereas H3K27me3 expanded into the FLT1 gene body exclusively in LECs (Fig. 2f). Likewise, the lymphatic markers PROX1, PDPN and FLT4, were devoid of the H3K27me3 marks and manifested a ‘gain’ of histone states in LECs, whereas they adopted either ‘unmarked’ or ‘bivalent’ states in BECs (Fig. 2g). CD34 exhibited ‘active’ histone composition in both BECs and LECs with similar levels of H3K4me3 enrichment at the promoter region.

Collectively, we found that the endothelial lineage markers present differential enrichment profiles of H3K4me3 and H3K27me3 histone composition in BECs and LECs. In particular, a large number of blood vascular markers manifest a more repressive histone state in LECs, implicating a potential epigenetic mechanism in the maintenance of lymphatic lineage specificity.

### Promoter hypomethylation accounts to a lesser extent for the differential transcriptomes

**Fig. 3:**
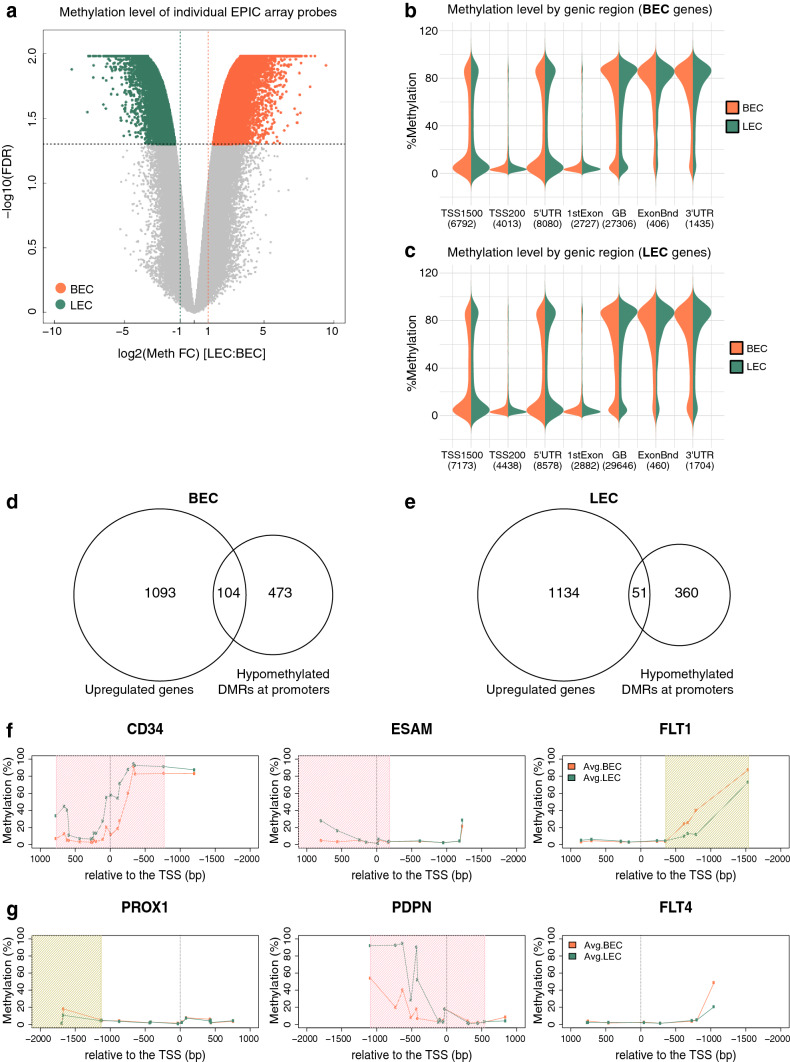
Elevated expression of endothelial lineage markers is not strictly controlled by DNA methylation of promoter regions. **a** Volcano plot representing the hypomethylated CpG loci in BECs (orange) and LECs (green), with a cutoff of log2 fold-change (FC) < 1 and adjusted *p* values < 0.05. **b** and **c** Violin plots showing the methylation levels at the indicated genic regions (TSS1500, TSS200, 5′UTR, 1st exon, gene body, exon boundaries and 3′UTR) of blood vascular- (**b**) and lymphatic-specific (**c**) genes in BECs (orange) and LECs (green). The numbers in bracket indicate the numbers of CpG loci included in each genic location. Venn diagrams showing the genes with upregulated expression and hypomethylated DMRs at their promoters in BECs (**d**) and LECs (**e**). **f** and **g** Detailed view of the methylation patterns at the promoters (− 2 kb to + 1 kb relative to the TSS at position 0) of the selected markers. The methylation levels at the interrogated CpG loci in BECs and LECs are denoted by connected dots in orange and green, respectively. The shaded regions highlight the DMRs which were hypomethylated in BECs (orange) and LECs (green)

Besides epigenetic regulations via histone modifications, DNA methylation is also of importance in regulating gene expression and cellular plasticity [27]. In fact, cells acquire specific DNA methylation patterns during the process of differentiation [28], where heavily methylated promoter regions in principle correlate with repressed transcription [29]. To investigate the potential relevance of DNA methylation in maintaining endothelial lineage specificity, we next examined the EC methylomes using the Infinium MethylationEPIC array. We observed a strong correlation between the methylation profiles of the biological replicates (Fig. S3a, b). Differential methylation analysis identified 19,139 loci (2.4% of probes) in BECs and 10,687 loci (1.3% of probes) in LECs to be hypomethylated in the respective cell type (Fig. 3a). It is notable that 4917 hypomethylated loci detected in BECs and 2751 in LECs lay within enhancer regions, constituting about 26% of the differentially methylated probes (Fig. 3a, Fig. S3c, d). To integrate the DNA methylation profiles with the transcriptomic landscape, we dissected the methylation patterns of differentially expressed genes, in accordance with the genic locations from upstream promoter regions to the 3′ untranslated regions (UTRs) (Fig. 3b, c). Although the upregulated genes primarily had a lowly methylated state around the promoters in the respective cell type, we did not observe a major shift in the methylation status in the opposing cell type at any genic region (Fig. 3b, c). In addition to interrogating separately the methylation levels of individual CpG loci, we also evaluated the variations in differentially methylated regions (DMRs) comprised of multiple consecutive CpG sites. Overall, there was only a modest agreement between increased expression and hypomethylation of the corresponding promoter in BECs (104 genes; Fig. 3d) and LECs (51 genes; Fig. 3e, Supplementary Table 2). Hypomethylated DMRs residing in the promoter regions were detected for CD34 and ESAM in BECs, and for PROX1 in LECs, in line with their increased expression in the respective cell type. Unexpectedly, the FLT1 promoter was hypermethylated in BECs compared to LECs. Likewise, the PDPN promoter was more heavily methylated in LECs whereas the promoter region of FLT4 displayed comparable methylation patterns in BECs and LECs (Fig. 3f, g).

### BCL6 and MEF2C as key transcription regulators potentially governing endothelial cell identity

**Fig. 4:**
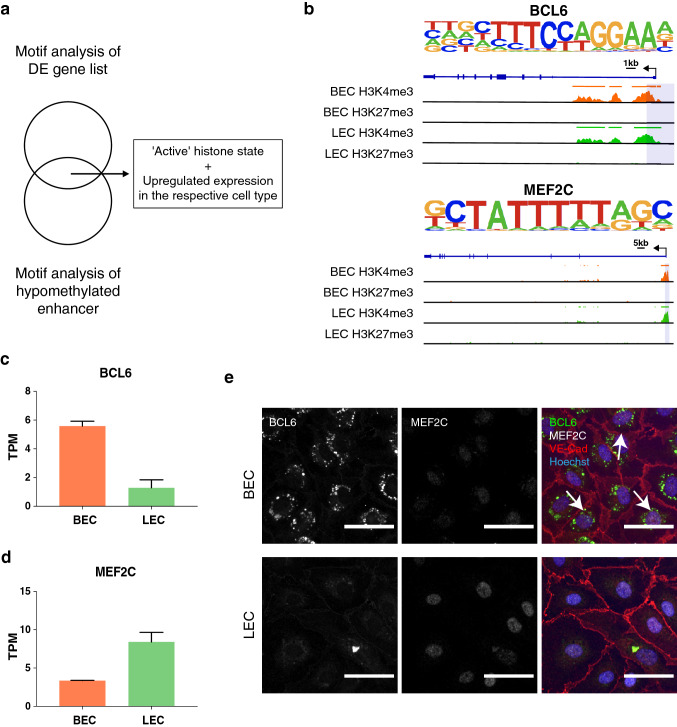
Motif analysis reveals two potential transcription factors important for endothelial cell lineage identity. **a** Workflow of motif analyses and the transcription factor selection criteria. **b** The enriched binding motifs of BCL6 and MEF2C, as well as their histone composition in BECs and LECs, respectively. Colored bars at the top of the signal tracks indicate the called ChIP-Seq peaks. Differential expression pattern of the transcription factors, BCL6 (**c**) and MEF2C (**d**), in BECs and LECs. **e** Representative immunofluorescence microscopy images of BCL6 and MEF2C in BECs and LECs (*n* = 2/cell line/donor). White arrows indicate BCL6-positive nuclei. Scale bar: 50 µm

Enhancers serve as the docking sites for transcription factor binding, and cell type-specific enhancers play an indispensable role in lineage determination [30]. In particular, DNA methylation levels of intronic enhancers have been shown to inversely correlate with gene expression across tissues and species [31]. Based on the differential methylation analysis, we identified more than 600 DMRs located in enhancer regions, which might represent potential regulatory elements involved in the patterning of lineage-specific transcriptomes. Of note, we found hypomethylated DMRs at the intronic enhancer regions of a handful of markers differentially expressed in BECs versus LECs (Fig. S3e, f, Supplementary Table 2). To gain an integrative view of epigenetic alterations and the differential transcriptomic profiles required for maintenance of lineage-specific EC identity, we next performed transcription factor binding motif analyses. We first selected the hypomethylated enhancer DMRs lacking H3K27me3 coverage (Supplementary Table 3), which were then used in parallel with the upregulated genes as inputs to search for enriched motifs (summarized in Supplementary Table 4). Subsequently, we assessed the histone states and the expression levels of the transcription factors identified by both motif analyses (Fig. 4a). We identified the transcription repressor BCL6 and the transcription enhancer factor MEF2C as potential key upstream regulators in BECs and LECs, respectively (Fig. 4b). Both transcription factors showed ‘active’ histone states (Fig. 4b) with increased RNA expression (Fig. 4c, d, Fig. S4a, b) and protein levels (Fig. 4e, Fig. S4c, d) in the respective cell type, suggesting their contribution to the transcriptomic and epigenetic regulation of endothelial lineage specificity. Most intriguingly, we observed differential expression patterns of certain reported interacting partners of BCL6 and MEF2C, including class II histone deacetylases (HDAC4 and HDAC9), SOX18 and KLF4, which were upregulated in the respective cell type (Fig. S4e) [32–35].

### Treatment with epigenetic drugs selectively promotes expression of blood vessel markers in lymphatic endothelial cells

**Fig. 5:**
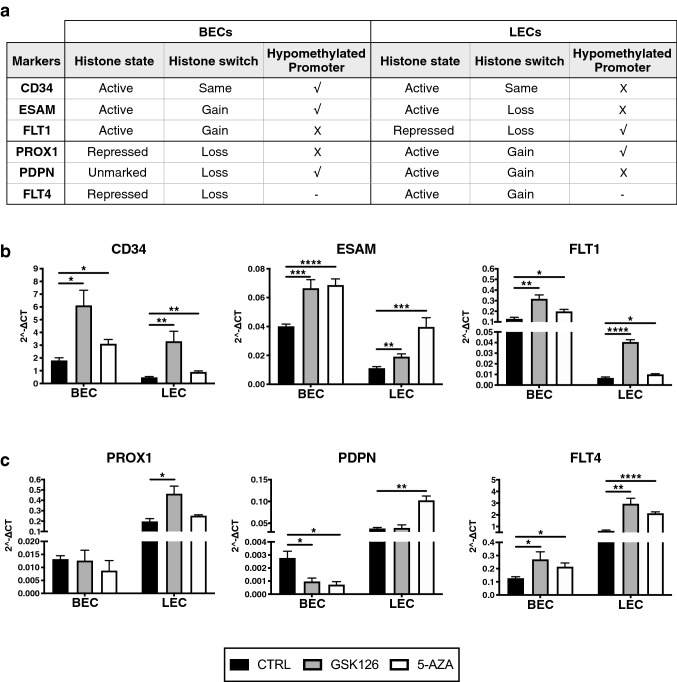
Induction of blood vascular endothelial cell markers by epigenetic inhibitor treatment of lymphatic endothelial cells. **a** Summary table of the epigenetic landscape of the endothelial lineage-specific markers. After treatment for 7 consecutive days with GSK126, 5-AZA or DMSO (CTRL), BEC (**b**) and LEC (**c**) marker genes were quantified by qPCR and shown as relative expression level using the 2^−ΔCT^ method. Data represent mean + SD. Significance was determined by unpaired *t* test (*n* ≥ 3 replicate/group, representative of 3 independent experiments), *p < 0.05; **p < 0.01; ***p < 0.001; ****p < 0.0001

We focused our attention on the epigenetic profiles of 3 BEC and 3 LECs markers, all of which showed an ‘active’ histone state in the respective cell type. Notably, these lineage-specific markers adopted a more favorable histone composition and/or a hypomethylated promoter compared to the opposing cell type (Fig. 5a). To further investigate the relevance of these epigenetic modifications for endothelial lineage specificity, we treated BECs and LECs with epigenetic drugs. More in detail, cells were treated with inhibitors targeting EZH2 (GSK126), a histone methyltransferase responsible for the formation of the repressive H2K27me3 histone mark (Fig. S5a), or DNA methyltransferases (5-AZA) that catalyze DNA methylation (Fig. S5b) [36]. We then studied the expression levels of CD34, ESAM and FLT1, as well as the lymphatic markers PROX1, PDPN and FLT4. The inhibition of H3K27 trimethylation by GSK126 strongly increased the expression levels of the blood vascular markers CD34, ESAM and FLT1 in LECs and to a lesser degree also in BECs (Fig. 5b). Likewise, DNA demethylation by 5-AZA treatment was also able to induce BEC marker expression in both cell types (Fig. 5b). In LECs, the expression of PROX1 and FLT4 was strongly increased by GSK126 treatment (Fig. 5c). On the other hand, 5-AZA treatment further enhanced the expression of PDPN and FLT4 in LECs (Fig. 5c). It is of interest that neither the blockade of H3K27 trimethylation nor inhibition of DNA methylation was able to upregulate the lymphatic markers PROX1 and PDPN in BECs, whereas we only observed a slight but significant increase in FLT4 expression (Fig. 5c).

Taken together with the histone enrichment and DNA methylation analyses, our findings suggest that LECs possess a more plastic phenotype compared to BECs. While blood vascular lineage markers were switched off during lymphatic specification, possibly via several epigenetic mechanisms, they could be reactivated in LECs by removing the repressive H3K27 trimethylation or DNA methylation. In contrast, BECs were more resistant to express lymphatic lineage markers, with the exception of FLT4 which was once expressed by the blood vasculature during embryonic development.

## Discussion

The lymphatic and blood vascular systems play fundamental roles in maintaining circulatory homeostasis, and impairment of their functions is associated with a large number of diseases, including cancer and chronic inflammatory diseases [37–39]. ECs possess unique transcriptional profiles that determine their lineage-specific functions. Considering the increasingly recognized importance of epigenetic alterations in cellular differentiation processes, the epigenome may also play an important role in preserving the identity of terminally differentiated ECs. Our study provides the first comparative epigenome-wide profiling of human primary ECs and reveals distinctive epigenetic signatures of BECs and LECs.

The distribution of methylation across the genome may vastly impact transcriptional activity, where hypermethylation of promoter regions is frequently associated with gene silencing [40]. Previously, a comparative DNA methylation analysis of BECs and LECs reported over 30,000 differentially methylated CpG sites, among which about 5% coincided with differentially expressed genes [20]. Similarly, we identified roughly 30,000 CpG loci that were differentially methylated between the two EC types. Despite multiple differences in the experimental setups, two-thirds of the differentially expressed genes in BECs and LECs identified by Bronneke et al. could also be confirmed in the present study (data not shown) [20]. Similarly, more than half to two-thirds of differentially methylated probes identified by our analysis were congruently found in the above-mentioned study (data not shown) [20], indicating an overall consensus between both studies regarding gene expression and DNA methylation of cultured human dermal ECs.

When we evaluated the DMRs present in the promoters or intronic enhancers, there was only a small number of genes that adopted both increased gene expression and hypomethylated promoters or intronic enhancers. Nevertheless, the bisulfite conversion of DNA employed in our study does not discriminate between cytosine methylation and hydroxymethylation. DNA hydroxymethylation is tightly linked to gene expression [41]. One of the dioxygenases that catalyzes the conversion of methylated cytosines into hydroxymethylcytosine (5hmC) is the Tet Methylcytosine Dioxygenase 1 (TET1). Interestingly, TET1 was differentially expressed between LECs and BECs (Fig. 1d). It is thus of great interest to investigate in further studies the important differences in DNA hydroxymethylation between the two cell types. On the other hand, by exploiting ChIP-Seq profiling of H3K4me3 and H3K27me3 histone marks, we found that over one third of BEC-specific genes showed a more ‘repressed’ histone state in LECs. In contrast, only 9.2% of genes upregulated in LECs could be ascribed to a ‘gain’ of histone states, with 10.2% even labeled with a ‘loss’ of favorable histone compositions. This suggests that the suppression of genes related to BEC identity, via the tuning of histone modifications, is crucial to the maintenance of the lymphatic lineage.

The induction of PROX1 expression during early embryonic development is considered to be the key driver of lymphatic fate determination [3, 42]. It has previously been reported that PROX1 acts as a binary switch by suppressing BEC lineage-specific genes [19]. Conversely, LECs could be partially reprogrammed into BECs when this brake was released [43]. Our study reveals that PROX1 expression is tightly regulated at the level of epigenetic modifications, by displaying a more ‘active’ histone state and a hypomethylated promoter in LECs. The finding that epigenetic drug treatment did not induce the expression of PROX1 in BECs reflects the importance of the lineage-specific expression of this master regulator of lymphatic specification. Similarly, PDPN expression could not be induced by 5-AZA and GSK126 treatment of BECs, in agreement with the major biological function of PDPN in promoting platelet aggregation, coagulation and thrombus formation when in contact with blood [44, 45]. FLT4 expression has been previously reported to be upregulated in blood vessels of tumors and healing wounds [11, 12, 46, 47]. Importantly, the level of FLT4 was elevated in BECs after the inhibition of DNA or histone methyltransferase, which may be in agreement with the developmental derivation of LECs from BECs, also indicating potential mechanisms underlying the aberrant expression of FLT4 in pathological conditions. In spite of the differential DNA methylation and histone composition profiles, all three BEC markers were upregulated in LECs by 5-AZA and GSK126 treatments, indicating the plasticity of LECs in adopting a blood vessel-like phenotype via epigenetic remodeling.

Besides the broad coverage over promoter regions, the Infinium MethylationEPIC array also features an additional coverage of intergenic and intragenic enhancer CpG loci. Tissue-specific DNA hypomethylation at enhancer regions is crucial for enhancer activity and strongly associates with marker expression [29, 48–50]. By conducting a meta-analysis of the three profiling techniques performed in this study, searching for potential upstream regulators using the hypomethylated enhancer regions together with the differential gene expression patterns and histone composition, we identified two transcription factors that potentially govern EC identity: the transcriptional repressor BCL6 (B-cell lymphoma 6) in BECs and the transcription enhancer factor MEF2C in LECs. BCL6 has previously been reported to be expressed by ECs [51] and to suppress angiogenic sprouting via the NOTCH signaling pathway [52]. Nevertheless, its role in the maintenance of the blood vascular endothelial lineage has not been described thus far. Notably, several of its interaction partners belong to the family of histone deacetylases (HDACs) that are essential for chromatin remodeling [34]. Thus, it would be of interest to dissect the participation of this transcriptional complex in monitoring the BEC epigenome in future studies. The lymphatic transcription regulator MEF2C belongs to the myocyte enhancer factor-2 (MEF2) family of MADS-box transcription factors that is often expressed in skeletal muscle cells [53]. Of note, MEF2C expression is indispensable for embryonic vascular development since its targeted deletion results in severe vascular defects and even lethality in mice [54]. The SOX18/KLF4-PROX1 axis is fundamental for VEGF-C/VEGFR-3 (FLT4) signaling and for the transdifferentiation of lymphatic progenitor cells from the cardinal veins during embryonic development [55, 56]. As studies have shown that MEF2C directly influences the transcriptional level of KLF4 [57] and the DNA-binding activity of SOX18 [58], it is plausible to speculate that MEF2C might also be involved in maintaining the lineage specificity of LECs and in shaping the lymphatic epigenome, potentially via interaction with HDACs [59].

The use of cultured cells in the current study may represent a limitation when performing transcriptomic and epigenetics analyses. In fact, previous studies have reported that cultured human and mouse ECs alter their gene expression profiles as compared to their native state in situ [60–62], along with major changes in epigenetic modifications upon culturing conditions [62, 63]. Nevertheless, our study, in agreement with those by Wick et al. and Amatschek et al. [60, 61], revealed that several of the major lineage-specific endothelial markers maintained their expression profiles in vitro*,* and were finely regulated by DNA methylation and histone modifications. These findings indicate that such an epigenetic memory is crucial for the maintenance of endothelial lineage specificity and function. In addition, LECs possess a more plastic phenotype compared to BECs, as highlighted by the resistance of BECs to upregulate the two key lymphatic-specific functional genes PROX1 and PDPN after epigenetic drug treatments. It will be of great interest to investigate epigenetic modifications of these distinct vascular beds in vivo under chronic pathological conditions such as inflammatory diseases and cancer.

## Materials and methods

### Cell culture

Cells were isolated as previously described [18]. Briefly, human neonatal foreskins were obtained after routine circumcisions. After enzymatic digestion, the epidermis was removed and dermal cells were mechanically released. CD34+ BECs were isolated and purified using an anti-human CD34 antibody (BD Pharmingen) conjugated to immunomagnetic beads (Dynal, Invitrogen). Thereafter, the remaining CD34− cells were incubated with immunomagnetic beads-conjugated anti-human CD31 antibody (Dynal, Invitrogen) to select for LECs. Cells were cultured under standard culture conditions (37 °C and 5% CO_2_) on collagen type-I (Advanced BioMatrix)-coated dishes (50 µg/mL) in EBM medium (Lonza) containing 20% FBS (Gibco), 1% penicillin/streptomycin (Gibco), 2 mmol/L l-glutamine (Gibco) and 10 µg/mL hydrocortisone (Sigma-Aldrich). LEC culture medium was supplemented with 25 µmol/mL cAMP (Sigma-Aldrich), while for BEC culture medium, endothelial cell growth supplement (PromoCell) was added, as previously described [18]. Cells were passaged every 5 days, sub-confluent cells between passage 6 and 7 were used for all experiments. All cells were routinely tested for mycoplasma contamination using the MycoScope PCR Mycoplasma Detection Kit (Genlantis).

### Flow cytometry

Endothelial cells were detached, washed with FACS buffer (DPBS with 2% FBS and 1 mM EDTA), and stained with Alexa647-conjugated mouse anti-human podoplanin (1:70, clone 18H5, NB600-1013AF647, Novus Biologicals) and PE-conjugated mouse anti-human CD31 antibodies (1:20, clone WM59, BD Pharmingen) in FACS buffer for 30 min at 4 °C. After a wash with FACS buffer, endothelial cells were acquired on a Cytoflex S (Beckman Coulter). Analysis was performed using the FlowJo software v10.5.3 (BD Biosciences).

### RNA sequencing

RNA was isolated from donor-matched BECs and LECs at passage 6 using the NucleoSpin RNA kit (Macherey–Nagel) according to the manufacturer’s instructions. From 100 ng of high-quality RNA, cDNA libraries were prepared and subjected to paired-end (100 bp) sequencing for 50 million reads per sample on average. Reads were aligned to the iGenomes UCSC hg19 build using STAR v2.4.2a [64] and blacklisted regions were removed by BEDTools v2.25.0 [65]. Mapped reads were assigned to expression counts using the iGenomes annotation file and featureCounts from the *Rsubread* package (v1.26.1) [66]. All meta-analyses were centered on the genomic coordinates of expressed genes (Transcripts Per Million; TPM > 0). Differentially expressed genes were defined by *DESeq2* (v1.16.1) [67] with the cutoff of false discovery rate (FDR) < 0.01 and log2 fold-change (log2FC) > 1. Pearson correlation coefficients between biological replicates were computed using normalized counts. A heatmap of differentially expressed epigenetic modifiers was generated with *ggplot2* (v3.2.0).

### RNA isolation, reverse transcription, and qPCR

Cultured LECs and BECs were lysed and RNA was extracted using the Nucleospin RNA kit (Macherey–Nagel) according to manufacturer’s instructions. RNA concentrations were measured using a NanoDrop ND-1000 spectrophotometer (Witec) and retrotranscribed using the High-Capacity cDNA Reverse Transcription Kit (Applied Biosystems). Gene expression in BECs and LECs was measured by qPCR using the PowerUp SYBR green master mix (Thermo Fisher) on a QuantStudio 7 Flex system. GAPDH served as an internal control. Relative expression of genes was calculated according to the 2^−ΔCT^ formula. Primer sequences were: CD34-fwd: TCC CAA AAG ACC CTG ATT GC; CD34-rev: AAT AGC CAG TGA TGC CCA AGA; ESAM-fwd: CAC CAG CAT TAG ATG TCA TCC ESAM-rev: CCT TGC AGA CAT AGA CTC CA; FLT1-fwd: CCC TTA TGA TGC CAG CAA GTG; FLT1-rev: CCA AAA GCC CCT CTT CCA A; PROX1-fwd: ACA AAA ATG GTG GCA CGG A; PROX1-rev: CCT GAT GTA CTT CGG AGC CTG; PDPN-fwd: CAG TTG AGA AAG ATG GTT TGT C; PDPN-rev: GAT GAT TGC ACC AAT GAA GC; FLT4-fwd: TCT GCT ACA GCT TCC AGG TGG; FLT4-rev: GCA GCC AGG TCT CTG TGG AT; MEF2C-fwd: GAA CGT AAC AGA CAG GTG AC; MEF2C-rev: CGC AAT CTC ACA GTC ACA C; BCL6-fwd: GAT GAG ATT GCC CTG CAT TT; BCL6-rev: TTC TTC CAG TTG CAG GCT TT; GAPDH-fwd: GAA ATC CCA TCA CCA TCT TCC AGG; GAPDH-rev: GAG CCC CAG CCT TCT CCA TG.

### ChIP-sequencing

BECs and LECs were fixed for 8 min at room temperature using 1% of formaldehyde. The ChIP-Seq experiment was conducted by the Diagenode ChIP-Seq Profiling service (G02010000, Diagenode). Chromatin was prepared using the iDeal ChIP-Seq kit for histones (C01010059, Diagenode). Chromatin was sheared using a Bioruptor Pico sonication device (B01060001, Diagenode) combined with a Bioruptor Water cooler for 12 cycles using 30″ [ON] 30″ [OFF] settings. Shearing was performed in 0.65 mL Bioruptor Pico Microtubes (C30010011, Diagenode) with 1 million cells in 100 μL. 50 μL of this chromatin was used to assess the size of the DNA fragments obtained by High Sensitivity NGS Fragment Analysis Kit (DNF-474) on a Fragment Analyzer (Advanced Analytical Technologies, Inc.).

ChIP was performed using an IP-Star®Compact Automated System (B03000002, Diagenode) following the protocol of the aforementioned kit. Chromatin corresponding to 1 million cells was immunoprecipitated using the following antibodies: H3K27me3 (C15410195, Diagenode) and H3K4me3 (C15410003, Diagenode). Chromatin corresponding to 1% was set apart as input. The DNA after reverse cross-linking was quantified using Qubit dsDNA HS Assay Kit (Q32854, Thermo Fisher Scientific). qPCR analyses were performed to check ChIP efficiency using KAPA SYBR FAST (Sigma-Aldrich) on a LightCycler 96 System (Roche). Libraries were prepared from input and immunoprecipitated DNA using the MicroPlex Library Preparation Kit v2 (12 indices) (C05010013, Diagenode). Library amplification was assessed using the High Sensitivity NGS Fragment Analysis Kit (DNF-474) on a Fragment Analyzer. Libraries were then purified using Agencourt AMPure XP (Beckman Coulter) and quantified using the Qubit dsDNA HS Assay Kit. Finally, their fragment size was analyzed by High Sensitivity DNA Analysis Kits on a 2100 Bioanalyzer system (Agilent). When the proportion of fragments > 1 Kb was too high, libraries were subjected to a double size selection with Agencourt AMPure XP and newly quantified and analyzed to assess their final size.

The libraries were subjected to single-read sequencing with at least 15 million reads per sample for input, 8 million reads for the H3K4me3 mark and 35 million reads for H3K27me3 modification. Sequencing reads were aligned to the hg19 genome using bowtie2 v2.2.3 [68], deduplicated with Picard v1.139 and filtered for blacklisted regions. The correlation coefficients between biological replicates of each histone mark were computed using deepTools v3.3.0 ‘multiBamSummary’ and heatmaps were generated with ‘plotCorrelation’ [69]. MACS2 v2.1.1 was used for calling broad H3K27me3 peaks (-broad -m 3 50 -broad-cutoff 0.01 -nomodel -extsize 260) and narrow H3K4me3 peaks (-m 7 50 -q 0.01 -nomodel -extsize 220), normalized signal was quantified as fragment per million reads [70]. Fold enrichment (bdgcmp-m FE) comparing histone occupancy against input controls was calculated. The independent signal tracks of biological replicates were combined using the unionbedg sub-command in BEDTools to generate an average track for visualization in the Integrative Genomics Viewer (IGV; v2.4.14) [71]. Peaks identified in both biological replicates were retained for downstream analyses. The distribution of histone enrichment peaks in either cell type across different genomic regions was assessed by the *ChIPseeker* package (v1.21.0) [72]. Enriched peaks were classified into promoter-overlapping (− 2 kb to + 1 kb relative to the TSS) or gene body-spanning regions, which were subsequently utilized for histone state grading of individual genes.

The *DiffBind* package (v2.13.0) was used to perform differential peak analysis [73, 74] with both DEseq2 and EdgeR [75, 76] methods. We filtered for differentially enriched peaks with a cutoff of fold enrichment > 1 and FDR < 0.05 in both methods.

### DNA methylation

DNA from BECs and LECs was extracted using the DNeasy Blood & Tissue Kit (Qiagen). DNA was bisulfite-converted and hybridized onto the Infinium MethylationEPIC BeadChip array that interrogates the methylation levels of over 850,000 CpG sites across the genome. With regard to the genomic context of the CpG sites, 34% of probes are located at the promoters, 39% of probes at intragenic CpGs and the remaining 26.9% of probes in the intergenic regions that include enhancers (each covered by 1–3 CpG sites) defined by the ENCODE and the FANTOM5 projects [77]. The import of raw data, filtering of probes with detection *p* values (< 0.01), background correction, Subset-quantile Within Array Normalization (SWAN) and genomic coordinate annotations (hg19) were performed with the minfi package (v1.28.3) [78]. Potentially cross-hybridizing probes (≥ 47 nucleotide off-target homology) and probes overlapping with known SNPs (minor allele frequency ≥ 5%) defined by both [79, 80] were also removed, resulting in 811,245 probes remaining for downstream analysis. The *β*-values [methylated/(unmethylated + methylated)], representing the proportional methylated signals, were used for graphical visualization and the *M*-values [*M* = log2(*β*/1 − *β*)] were used for testing statistical significance. Linear models were fitted for differential methylation analysis of individual probes (CpG sites) with the limma package (v3.38.3) [81], where Benjamini–Hochberg (BH) adjusted *p* values below 0.05 were considered significant. DMRcate (v1.18.0) was utilized to identify differentially methylated regions (DMRs), constituted of minimum 3 CpG sites with Stouffer transformed FDR below 0.05 [82]. DMRs were subsequently assigned to genes with an overlapping promoter region (− 2 kb to + 1 kb relative to the TSS). Enhancer DMRs were defined as regions comprised of or at least containing significantly differentially methylated probes targeting enhancers.

### Transcription factor binding motif analyses

Transcription factor binding motif analysis of upregulated genes was performed with HOMER (v4.10) ‘findMotifs.pl’ (-start -2000 -end 1000) [83]. In parallel, we used HOMER ‘findMotifsGenome.pl’ (-size given) for motif analysis of hypomethylated enhancer DMRs devoid of the H3K27me3 mark. The transcription factors yielded from both motif analyses were then filtered for their histone states and gene expression patterns, where those with an ‘active’ histone state and upregulated expression in the respective cell type were identified as potential upstream transcriptional regulators.

### Immunofluorescence stainings

Cells were seeded into wells of a 24-well plate containing glass slides and cultured until confluence. Cells were fixed with 4% PFA for 10 min at room temperature, blocked in blocking solution (PBS, 0.05% Tween 20, 0.1% Triton-X, 5% donkey serum, 5% BSA) and stained with primary antibodies (in blocking solution) overnight at 4 °C, followed by washing in PBS and incubation with secondary antibodies for 1 h at room temperature. After washing, glass slides were mounted using Mowiol (Sigma-Aldrich). Primary antibodies were: rabbit anti-MEF2C (1:400, Cell Signaling, 5030), mouse anti-BCL6 (1:100, Santa Cruz, sc-7388) and goat anti-VE-cadherin (1:100, R&D, AF938). Secondary antibodies were: donkey anti-goat AlexaFluor594, donkey anti-mouse AlexaFluor488, donkey anti-rabbit AlexaFluor647 (1:1000, all from Life Technologies). Confocal images were taken with an LSM780 microscope (Zeiss).

### Western blot

Passage and donor-matched LECs and BECs were lysed with lysis buffer (25 mM HEPES, 5 mM EDTA, 1% triton-X, 150 mM NaCl, 10% glycerol, complete protease inhibitor cocktail) and then centrifuged at 13,000 rpm for 20 min at 4° to collect the supernatant. Protein concentration of lysates was determined using the Microplate BCA protein assay kit (Thermo Fisher), according to the manufacturer’s instruction. Proteins were separated by SDS-PAGE on 4–12% NuPAGE Bis–Tris protein gels (Invitrogen) and transferred to a PVDF membrane (Immobilon-P, Millipore). Membranes were blocked in 5% milk in TBS + 0.1% Tween20, and incubated with primary antibodies (rabbit anti-MEF2C 1:1000, Cell Signaling, 5030; mouse anti-BCL6, 1:500, eBioscience, GI191E; rabbit anti-b-Actin 1:5000, Abcam, ab8227; rabbit anti-H3K27me3 1:1000, Diagenode, C15410195, rabbit anti-DNMT 1:1000, Cell Signaling, 5032) in 5% milk in TBS + 0.1% Tween20, followed by washes and incubation with secondary antibodies (goat anti-rabbit and goat anti-mouse both 1:5000, Dako, labeled with HRP). Signal was developed with ECL Prime (GE Healthcare) and imaged on a ChemiDoc imaging system (Bio-Rad).

### Perturbation of gene expression of lymphatic and blood vascular endothelial cells

Cells were seeded and treated the following day with 0.2 µM of GSK126 (xcessbio) or 5 µM of 5-Aza-2′-deoxycytidine (5-AZA; Sigma-Aldrich) for 7 consecutive days. Media were changed every other day with fresh media supplemented with the corresponding treatment. At the end of the treatment, cells were lysed and RNA was extracted for qPCR quantification as described above.

### Statistical analyses

Statistical analyses were performed using Prism v7.0a (GraphPad Software Inc.). Data are shown as mean + SD. To determine statistical significance, a 2-tailed, unpaired Student’s t test was performed. Differences were considered statistically significant at *p* < 0.05. Bioinformatic analyses were performed with Python v2.7.6 and R v3.4.0.

## Electronic supplementary material

Below is the link to the electronic supplementary material.

Supplementary file1 (DOCX 1500 kb)

Supplementary file2 (XLSM 69 kb)

Supplementary file3 (XLSM 59 kb)

Supplementary file4 (XLSM 84 kb)

Supplementary file5 (XLSX 17 kb)

## Data Availability

RNA-Seq (E-MTAB-8950), ChIP-Seq (E-MTAB-8951) and DNA methylation (E-MTAB-8952) data are accessible in ArrayExpress.
