# Supplementary material for: Epigenetic regulation of the lineage specificity of primary human dermal lymphatic and blood vascular endothelial cells
Source: Angiogenesis. 2020 Sep 12;24(1):67–82. doi: 10.1007/s10456-020-09743-9 (PMC7921079; doi:10.1007/s10456-020-09743-9)
Supplement: Supplementary file 1 — Supplementary file1 (DOCX 1500 kb) [file 10456_2020_9743_MOESM1_ESM.docx]

**Epigenetic regulation of the lineage specificity of primary human dermal lymphatic and blood vascular endothelial cells**

Carlotta Tacconi^1^*, Yuliang He^1^*, Luca Ducoli^1^, Michael Detmar^1+^

^1^Institute of Pharmaceutical Sciences, Swiss Federal Institute of Technology, ETH Zurich, 8093, Zurich, Switzerland

^+^Corresponding author:

Michael Detmar, M.D.

Vladimir-Prelog-Weg 3, HCI H303

8093 Zurich, Switzerland

Telephone: 0041 44 633 73 61

Fax: 0041 44 633 13 64

Email: [michael.detmar@pharma.ethz.ch](mailto:michael.detmar@pharma.ethz.ch)

* These authors equally contributed to this work

**
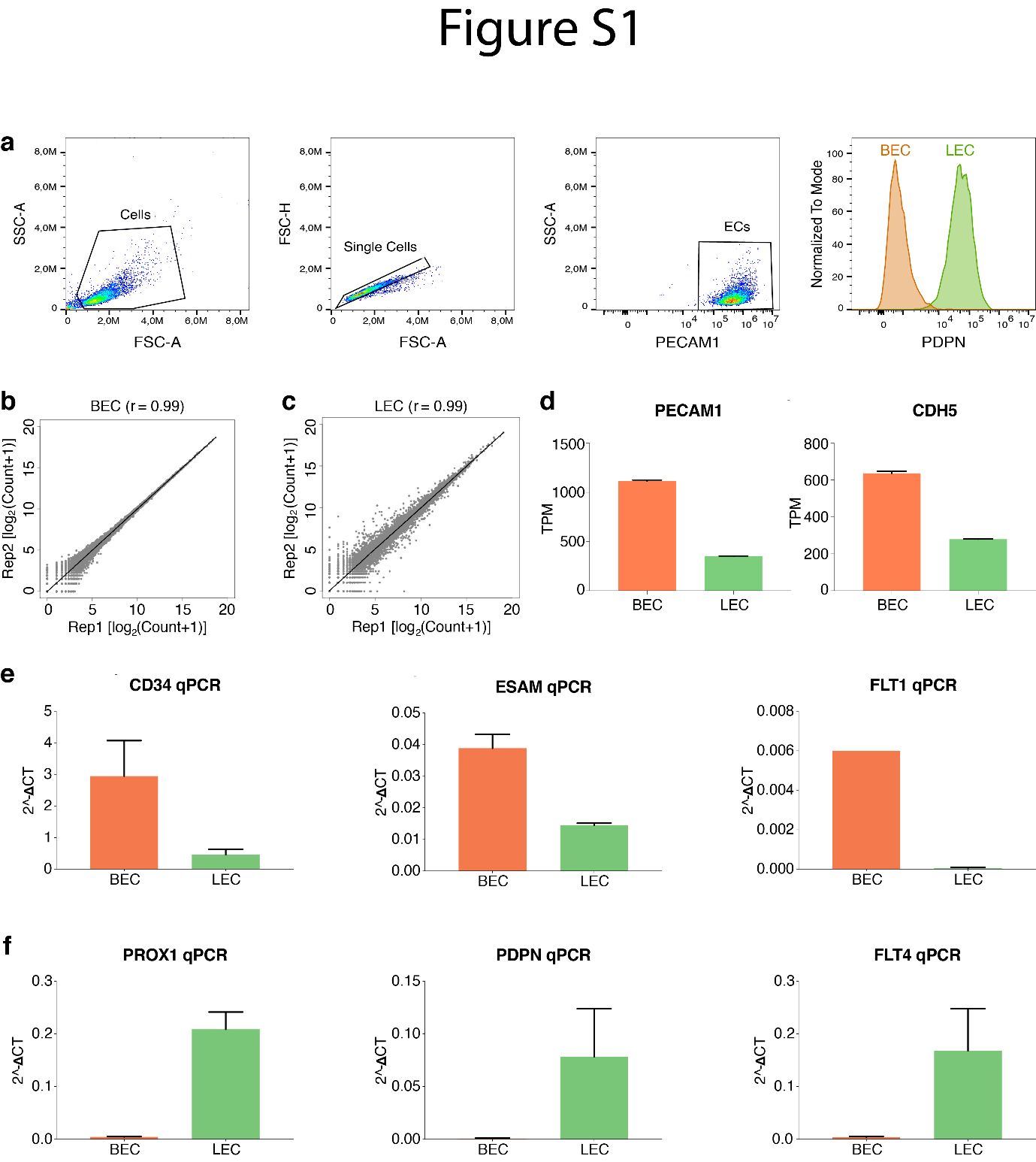
**

**Figure S1.** (a) Representative FACS gating strategy of cultured human neonatal dermal LECs and BECs showing PECAM1 and PDPN expression. Pearson correlation (r = 0.99, p < 2.2e-16) between normalized and log2-transformed RNA-Seq read counts from the two biological replicates of BECs (b) and LECs (c). (d) Expression levels (in TPM) of the pan-endothelial markers PECAM1 (CD31) and CDH5 (VE-cadherin). Expression levels of the lineage-specific BEC markers (CD34, ESAM and FLT1) in (e) and LEC markers (PROX1, PDPN and FLT4) in (f) quantified by qPCR (n = 2 independent cell lines). GAPDH was used as the housekeeping gene. Data shown as mean + SD.

**Figure S2.** Pairwise Pearson correlation heatmaps depicting ChIP-Seq signals between the two biological replicates of BECs (a) and LECs (b) across the entire genome. (c) Genomic distribution of ChIP-Seq peaks congruously detected in both biological replicates. Promoters were defined as the regions spanning 2 kb upstream of the TSS to 1 kb downstream.

**
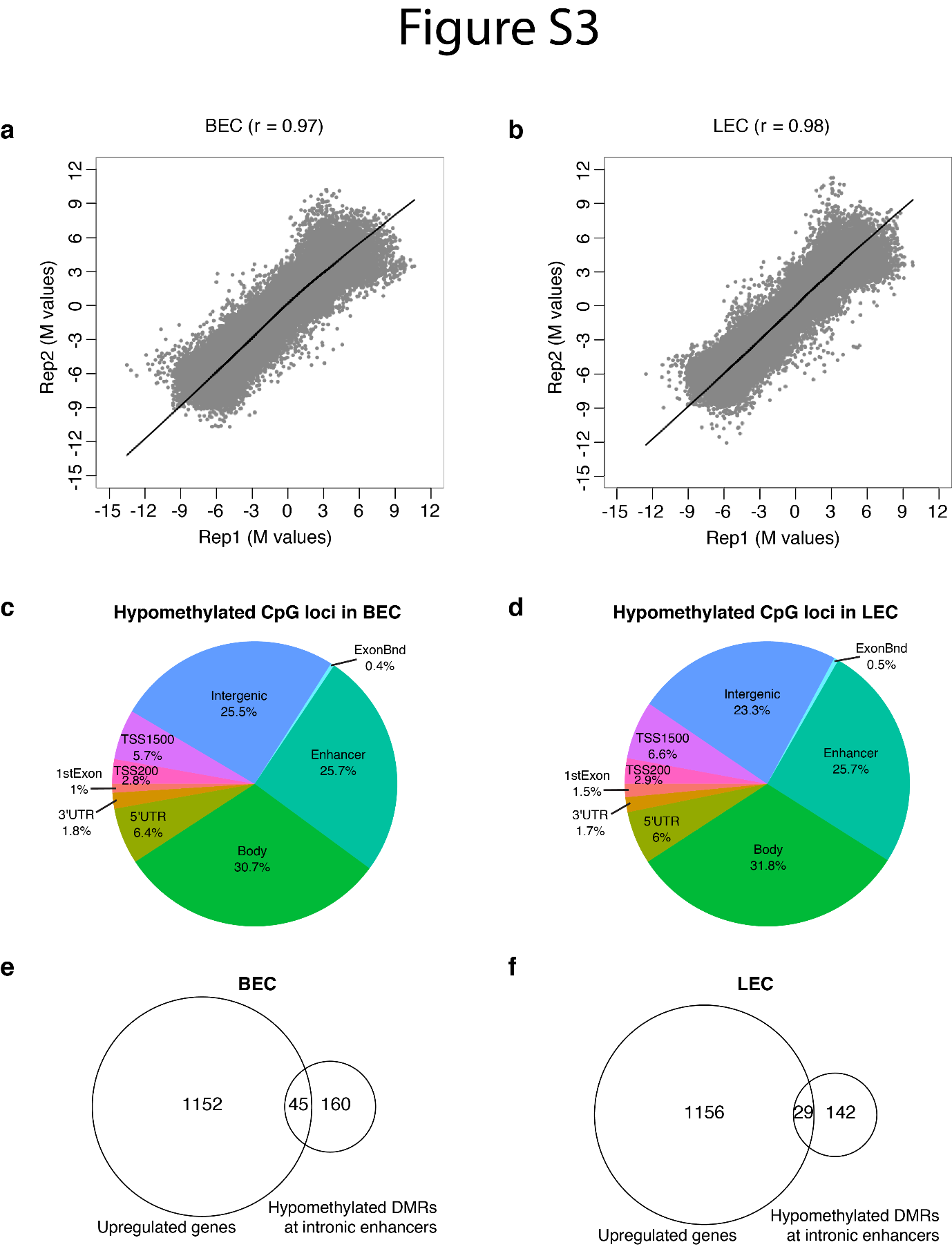
**

**Figure S3.** Correlation (Pearson, r = 0.97~0.98, p < 2.2e-16) between DNA methylation patterns, detected by the retained 811,245 probes, in the two biological replicates of BECs (a) and LECs (b). Values shown are log2-transformed M-values. Genomic distribution of hypomethylated CpG loci in BECs (c) and LECs (d). Venn diagrams showing the genes with upregulated expression and hypomethylated DMRs located at intronic enhancers in BECs (e) and LECs (f).

**
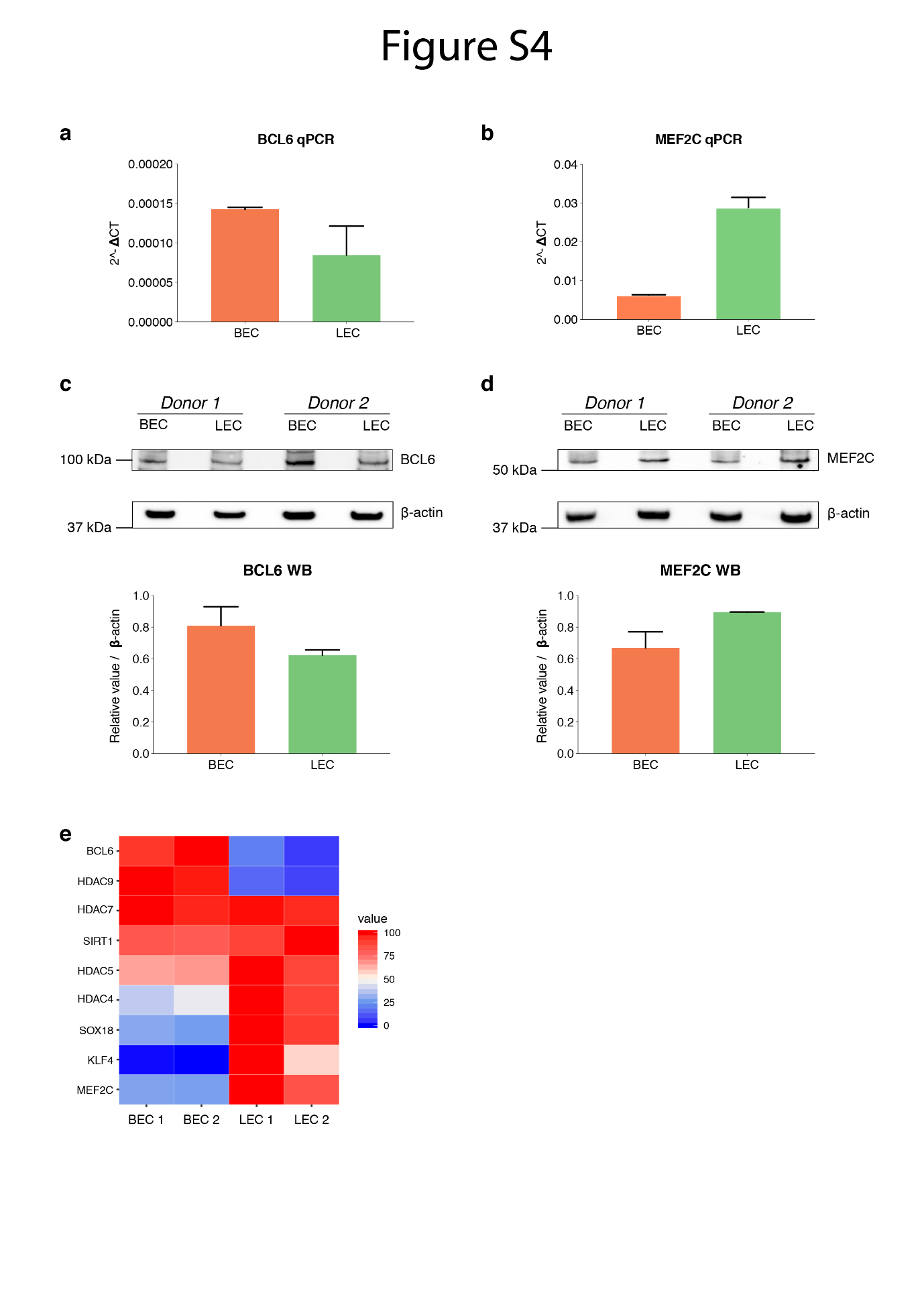
**

**Figure S4.** Gene expression levels (by qPCR) and protein levels (by western blots) of BCL6 (a and c) and MEF2C (b and d) in BECs and LECs. Data shown as mean + SD. (e) Heatmap showing the expression pattern of reported interacting partners of BCL6 and MEF2C in each cell type. Values are shown as the percentage of maximum expression of the respective gene.

**
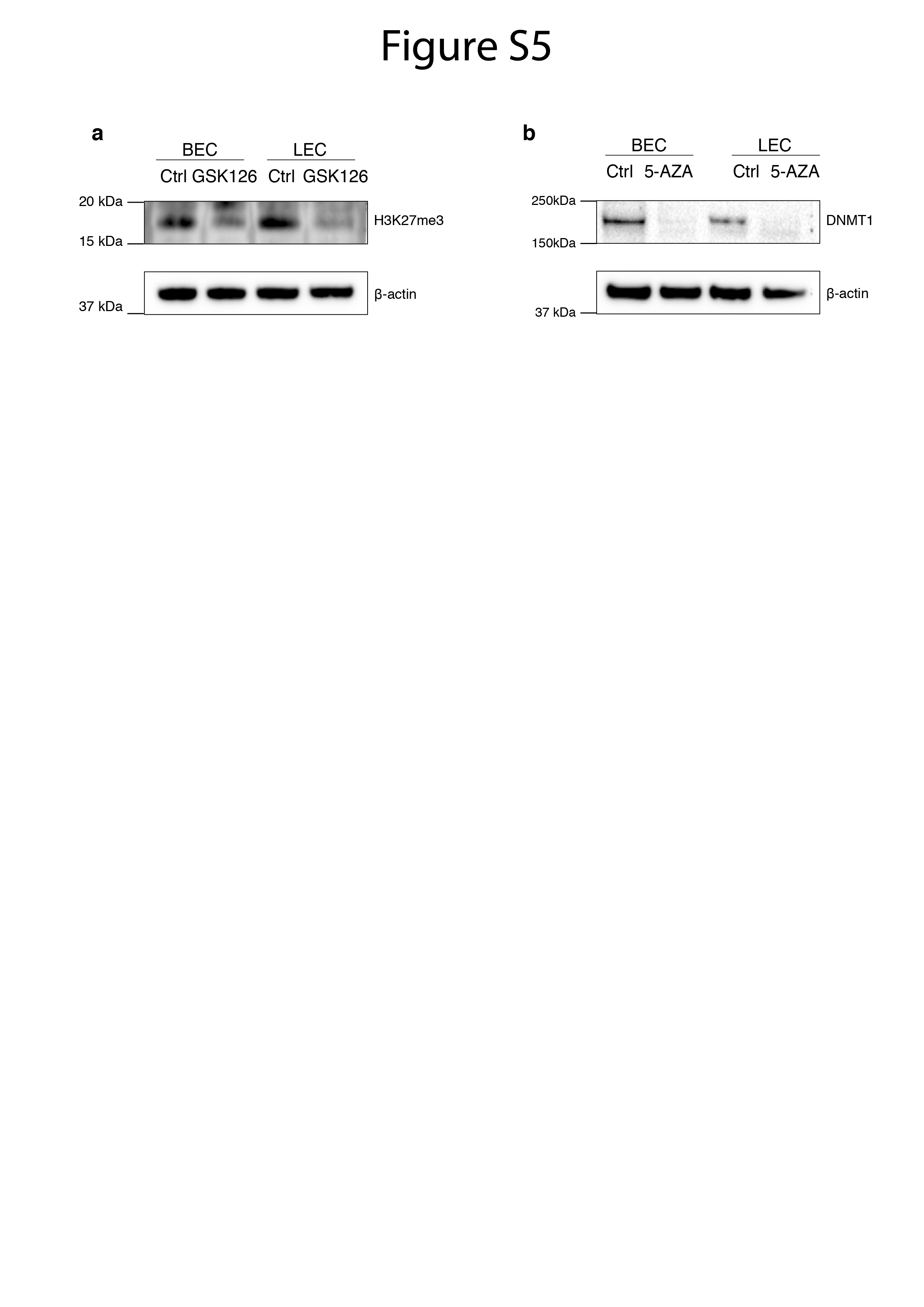
**

**Figure S5.** Protein levels (by western blots) of H3K27me3 (a) and DNMT1 (b) in BECs and LECs after 7-day treatment with GSK126, 5-AZA or DMSO control (Ctrl). β-actin was used as an internal protein loading control.

**Table S1.** Histone states of the genes differentially expressed in BECs and LECs.

**Table S2.** Consensus between promoter/intronic enhancer hypomethylation and upregulated expression in BECs and LECs.

**Table S3.** Hypomethylated enhancer DMRs in BECs and LECs devoid of H3K27me3 coverage.

**Table S4.** Transcriptional regulators identified by motif analyses using differentially expressed gene lists or hypomethylated enhancer DMRs devoid of the H3K27me3 mark, both with a cutoff of *p*-value ≤ 0.01. Transcription factors congruently identified by both analyses were highlighted in red.
